# Marjolin's Tumor Complicating Chronic Periprosthetic Infection of a Total Knee Arthroplasty

**DOI:** 10.7150/jbji.34679

**Published:** 2019-04-20

**Authors:** Timothy Horan, Matthew T. Wallace, Albert J. Aboulafia, Janet D. Conway

**Affiliations:** 1 Philadelphia College of Osteopathic Medicine Orthopaedic Surgery, Philadelphia, PA; 2 MedStar Georgetown Cancer Institute, National Center for Bone and Soft Tissue Tumors, MedStar Franklin Square Medical Center; 3 Rubin Institute for Advanced Orthopaedics, Baltimore, MD

## Abstract

Marjolin's tumor is a term used to describe a malignancy developing in the setting of a chronic wound, infection, or other tissue subject to chronic inflammatory changes. These malignancies usually present after many years of chronicity, and can range from lower grade basal cell carcinomas to high-grade sarcomas. We present the case of a squamous cell carcinoma that developed within a chronic periprosthetic infection of a total knee arthroplasty of 7 years duration. The intra-articular location, association with an orthopaedic implant, and brief latency period are all unique features of this case.

## Presentation

**Figure 1:**
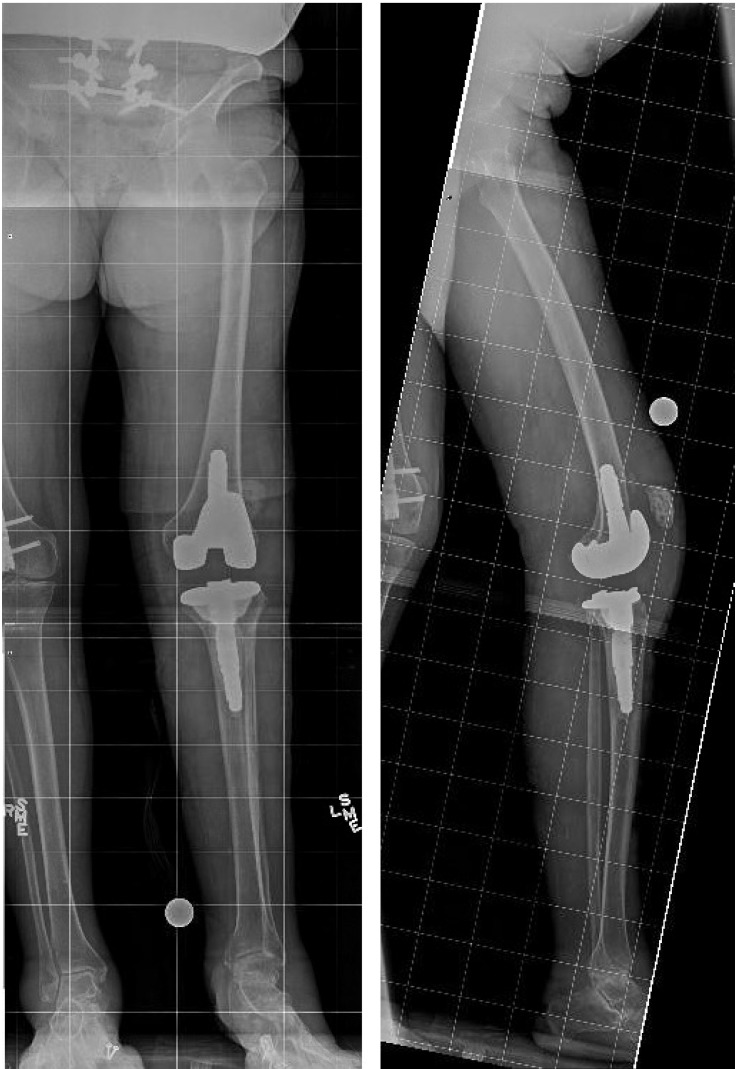
** 7/30/2013.** AP (A) and Lateral (B) radiographs at the time of initial consultation demonstrating a stemmed TKA with substantial effusion and patella alta.

A 57 year-old female with a past medical history of rheumatoid arthritis on long-term hydroxychloroquine and leflunomide, as well as pulmonary embolism and hypothyroidism, underwent left total knee arthroplasty (TKA) on 9/16/2009. She developed an acute hematogenously-seeded periprosthetic joint infection (PJI) of unspecified organism and underwent debridement and exchange of polyethylene on 6/7/2011, followed by 6 weeks of parenteral antibiotics. She was asymptomatic after this procedure until 6/4/2012, at which time she developed a small cyst over the knee which was excised on 12/3/2012. This was complicated by wound healing difficulties, necessitating a split-thickness skin grafting procedure, which was then followed shortly by a second PJI. She underwent a 2-stage revision with explantation of components and antibiotic spacer insertion on 3/8/2013, followed by spacer removal and implantation of components on 6/4/2013. After persistence of PJI was suspected, she was referred to a musculoskeletal infection specialist for consultation (Figure 1).

**Figure 2:**
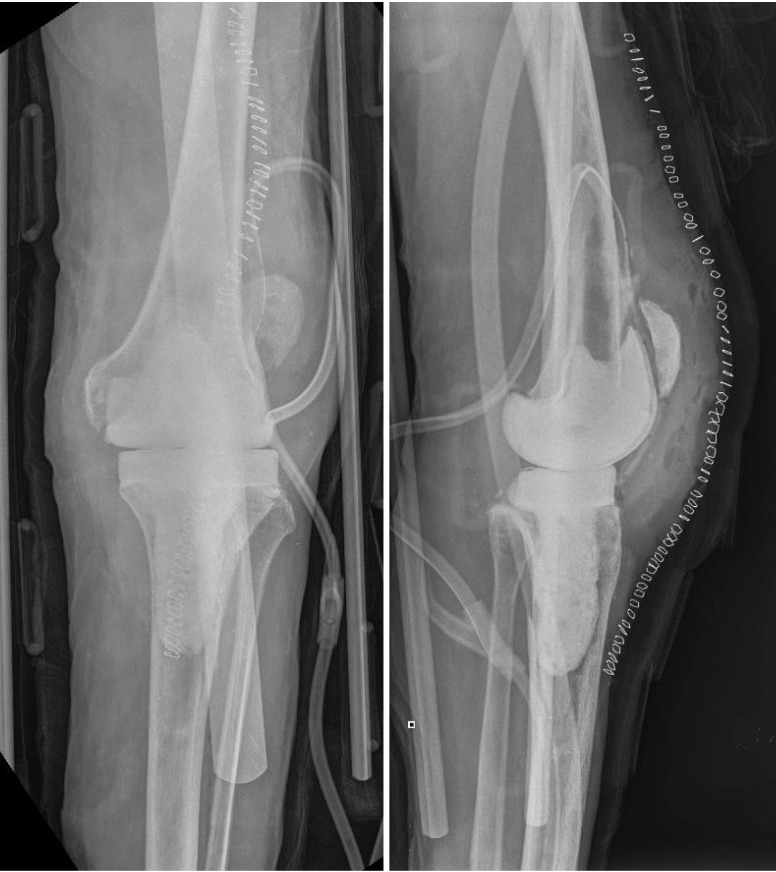
** 9/30/2013.** Postoperative AP (A) and Lateral (B) radiographs demonstrating appropriate alignment of the knee after explantation of components and PMMA antibiotic spacer insertion.

The patient underwent explantation of her TKA and insertion of a PMMA antibiotic spacer impregnated with vancomycin and tobramycin on 9/28/2013 (Figure 2). Intraoperative cultures were positive for Pseudomonas aeruginosa, and she was prescribed a course of IV aztreonam and PO ciprofloxacin for 6 weeks. Her postoperative course was complicated by a proximal tibial fracture, which was managed uneventfully in a long leg cast. A left knee aspiration and serologies performed on 12/10/2013 found no evidence of persistent infection.

**Figure 3:**
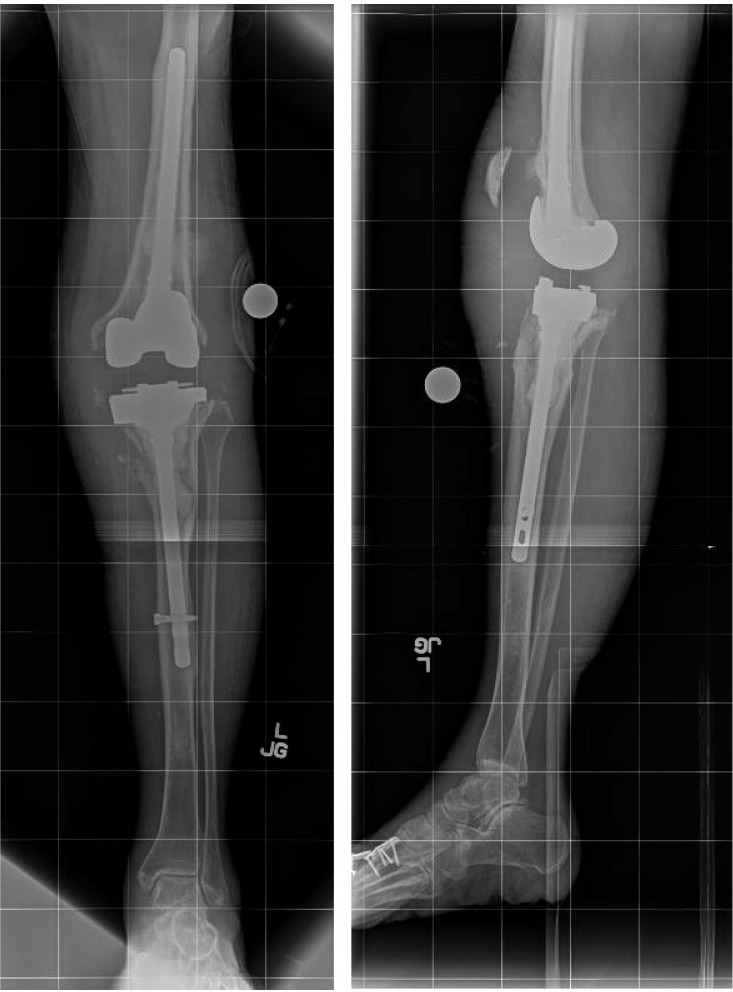
** 8/5/2014.** AP (A) and Lateral (B) radiographs demonstrating substantial effusion of the knee joint, and lucency and periprosthetic loosening around the stemmed tibial implant.

**Figure 4:**
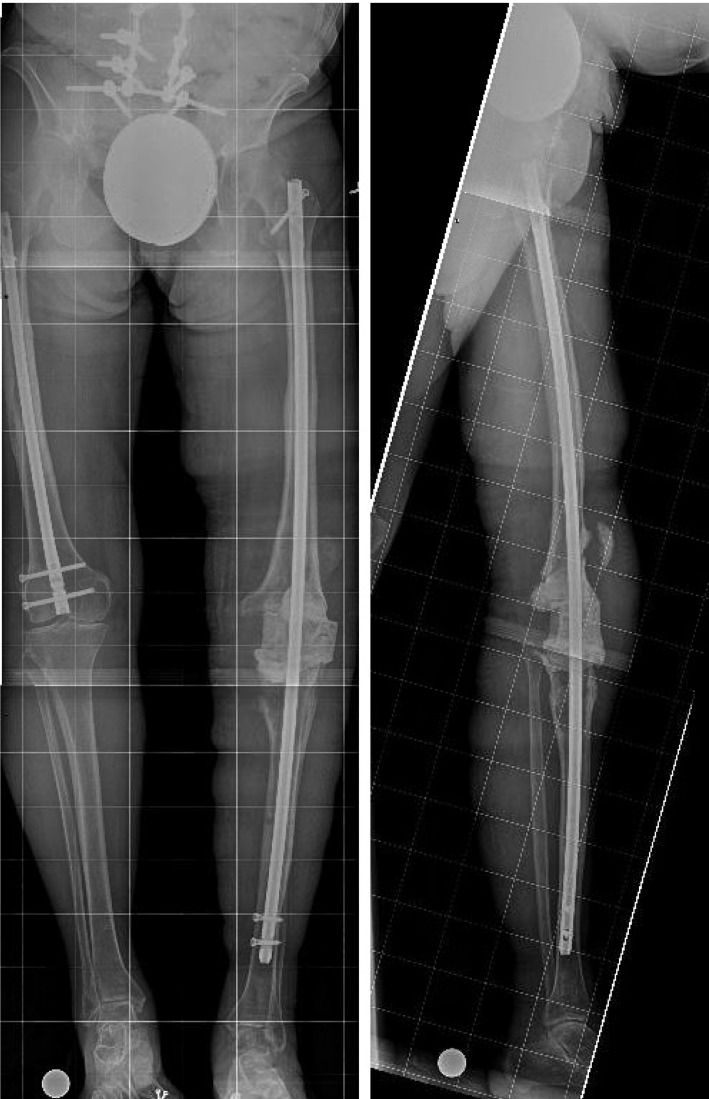
** 1/20/2015.** AP (A) and Lateral (B) radiographs demonstrating a locked lower extremity arthrodesis nail through a static antibiotic cement block.

Revision TKA with stemmed implants was performed on 2/6/2014 with 5 intraoperative frozen section pathology specimens demonstrating no evidence of infection or acute inflammation. Her postoperative course was complicated this time by a left lower extremity DVT despite therapeutic coumadin. After uneventful healing of her surgical wound, chronic venous stasis changes were observed in the lower extremity, managed with compression stockings. At a 6 month postoperative visit on 8/5/2014 the patient reported “popping” of her knee and radiographs demonstrated loosening around the tibial component (Figure 3). An aspiration obtained at this visit demonstrated 41,310 WBC's, 97% PMN's, negative crystals and growth of coagulase negative *Staphylococcus* in broth only. A repeat aspiration on 8/26/14 demonstrated 36,600 WBC's, 97% PMN's, no crystals and growth of coagulase negative *Staphylococcus*. Due to persistence of her infection now after two 2-stage revisions, it was recommended at this time that the patient undergo component explantation with a knee arthrodesis and an extended course of IV clindamycin and ciprofloxacin (Figure 4). This was performed on 12/15/2014. Surgical site pathology confirmed inflammatory debris.

The patient tolerated this reconstruction well over the course of 15 months, at which point she presented on 3/2/2016 with persistent extremity edema that had failed aggressive conservative management. An aspiration of the knee was negative at this time. Over the ensuing months the patient developed a 5cm x 3cm anterior tibial wound without drainage or fistula formation. Local wound care was performed without improvement in the area of ulceration. A technetium-99m bone scan obtained on 11/11/2016 demonstrated findings consistent with persistent osteomyelitis of the left anterior tibia. It was recommended that the patient undergo exchange of her arthrodesis nail and antibiotic spacer, followed by split-thickness skin grafting of her tibial wound, which she delayed until 5/18/2017 in favor of the patient's preference for observation. Intraoperative cultures were positive for *E. coli*, coagulase negative *Staphylococcus*, beta-lactamase positive *Bacteroides,* and *Enterococcus* group D. The patient was initiated on IV aztreonam, PO doxycycline and PO metronidazole. She continued to develop multiple anterior venous stasis ulcerations of the left lower leg, one of which required an additional split-thickness skin grafting on 11/16/2017 after failing conservative management.

**Figure 5:**
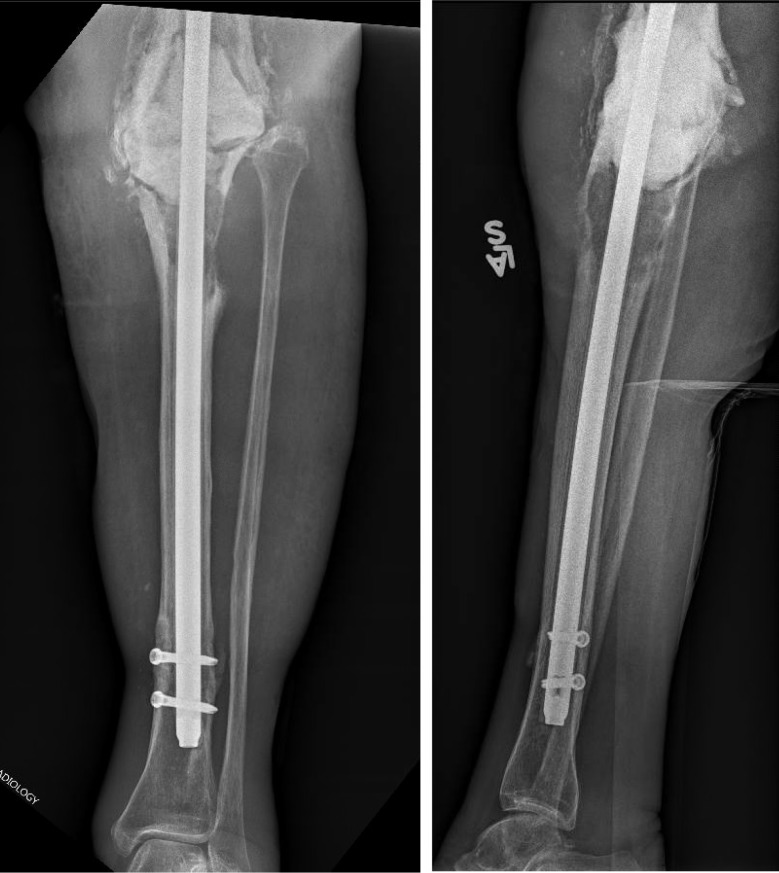
** 1/8/2018.** AP (A) and lateral (B) radiographs demonstrating progressive lysis of the proximal tibia below the PMMA antibiotic cement spacer.

The patient presented on 1/8/2018 with increasing erythema, warmth, and new skin ulcerations over the anterior left tibia. Cultures of the ulcerations demonstrated *Corynebacterium*. Her inflammatory markers at this time were elevated; erythrocyte sedimentation rate (ESR) was 101 and c-reactive protein (CRP) was 87.2. Plain radiographs demonstrated progressive lytic changes of the tibia suggestive of abscess formation and osteomyelitis (Figure 5). A frank discussion was held regarding the surgical options for what was becoming an increasing compromised extremity. The patient was not receptive to a discussion of extremity ablation at this time, and after another period of observation, she agreed to undergo an additional exchange of her arthrodesis nail and antibiotic spacer.

**Figure 6:**
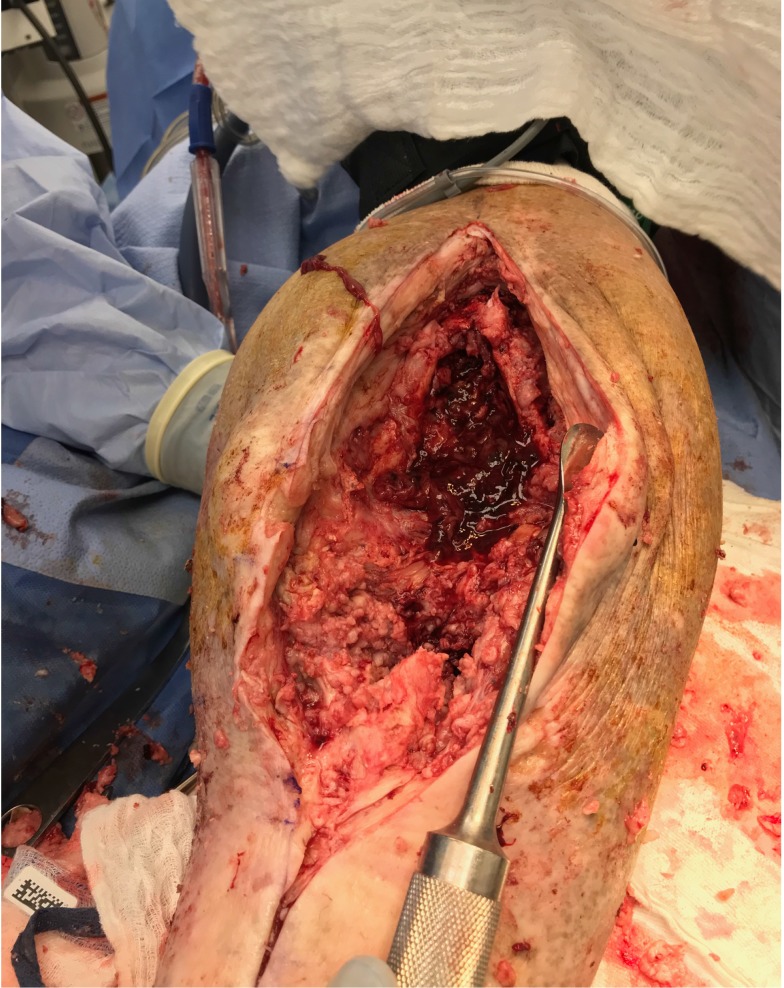
Intraoperative appearance of wound demonstrating diffuse nodular involvement of the joint.

**Figure 7:**
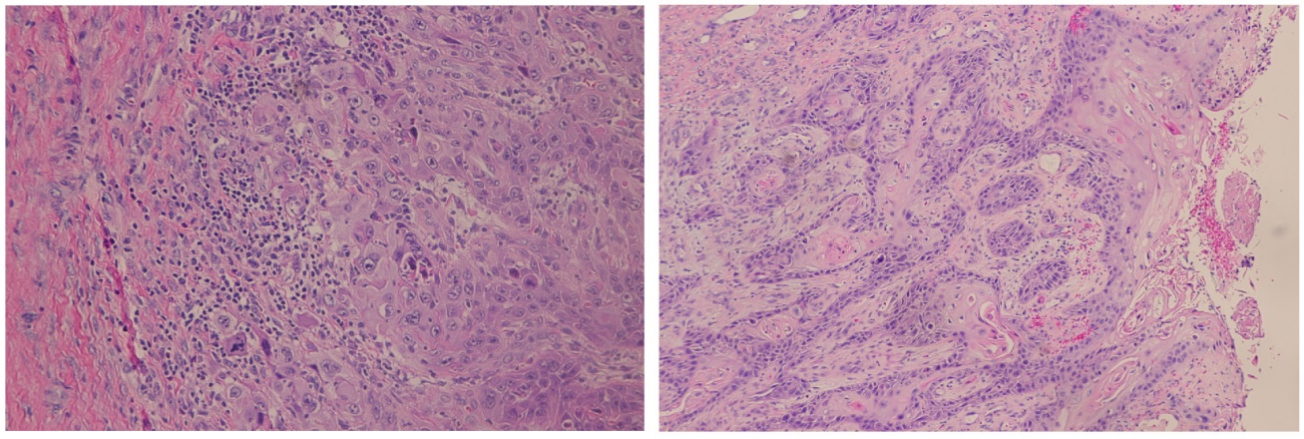
Hematoxylin and eosin histopathology at Low (100x) (A) and High (200x) (B) power imaging demonstrating squamous cell carcinoma, with nests of epithelioid cells and distinguishing keratinous debris

On 7/5/2018 the patient returned to the operating room for a planned exchange of her arthrodesis rod, irrigation and debridement, and reinsertion of antibiotic cement spacer. Intraoperative cultures grew 3 different strains of *E. coli*, Beta-lactamase positive *bacteroides*, and *enterococcus faecalis*. Upon exposure, the tissue bed around the proximal tibia and distal femur was observed to have an unusual, pebble-like appearance (Figure 6). Intraoperative frozen section pathology consultation was obtained, returning a diagnosis of squamous cell carcinoma (Figure 7). The wound was closed and an orthopaedic oncology consultation was obtained.

A staging CT of the chest abdomen and pelvis demonstrated an enlarged left inguinal lymph node without other signs of pulmonary or locoregional metastasis. Due to the extensive involvement and contamination of the joint with carcinoma, as well as the overall compromised nature of the extremity, ablation of the extremity was recommended with above knee amputation, combined with sentinel lymph node biopsy, which was performed on 7/9/2018. The sentinel lymph node, measuring 6.3 by 3.5 by 2.3 cm was negative for carcinoma, and consisted of an enlarged, reactive lymph node from chronic infection. Margins from the skin, soft tissue, and femoral canal were negative for malignancy. The patient was discharged with 2 weeks of oral doxycycline. She proceeded to heal her stump wound uneventfully. A restaging PET-CT performed 6 months postoperatively demonstrated persistent reactive inflammatory lymphadenopathy in the left inguinal region, with no evidence of metastatic disease.

## Discussion

The first observation of Marjolin's ulcer (MU) is credited to Aurelius Cornelius Celsus in the first century, who described malignant changes in a burn scar.^11^ The term “Marjolin Ulcer” was coined by Robert Smith in 1850, who described the pathologic transformation of chronic wounds into malignancy. Malignancies observed include squamous cell carcinoma (SCC), basal cell carcinoma (BCC), melanoma and sarcoma.^7^ Marjolin's Ulcer is a rare complication which has been observed in scar tissue, chronic ulcers, osteomyelitis, fistulas and any wound aggravated by persistent inflammation.^11^,^12^,^17^,^19^ The pathophysiology of chronic wounds undergoing malignant transformation has been debated over the past century and no specific factor has been identified.^15^ Prevailing theories suggest repetitive tissue damage leading to cellular proliferation, prone to spontaneous mutations. Necrotic tissue toxins may exacerbate mutations leading to inhibited apoptosis. This is likely from a multifactorial etiology including environmental, immunological and genetic factors summarized in Table 1.^11^,^13^,^14^,^15^,^16^

### Epidemiology

Marjolin's ulcer has been classically associated with burns, reported to occur as frequently in 2% of chronic burn scars.^14^ The most common malignancy observed as Marjolin tumors is squamous cell carcinoma (71%), followed by basal cell carcinoma (12%), melanoma (6%), sarcoma (5%), and other neoplasms (4%).^2^ The hallmark of Marjolin's tumor is the chronicity of the primary disease. The average latency period is 32 years, but acute cases have been reported with malignant transformation occurring within 12 months.^1^,^2^ Marjolin's ulcers most frequently affect the extremities, with a predilection for the lower extremities (53.3%), followed by the upper extremities (18.7%), torso (12.4%), and head and neck (5.8%).^2^ Metastatic disease is observed in 32% of patients with squamous cell carcinoma.^3^

### Diagnosis

Any chronic wound that undergoes a change in appearance or drainage should alert providers to the possibility of malignant transformation. A comprehensive history detailing the chronicity of the wound as well as attempted treatments should be documented. Suspect lesions should undergo biopsy performed by or at the direction of the treating surgeon.^10^ Tissue sampling, either by percutaneous needle or incisional biopsy, should be planned with the definitive resection in mind.^12^ After histologic confirmation of malignancy, staging by way of local MRI and body CT or PET scan are recommended to identify lymph node and distant metastases.^10^ Staging provides a reproducible way to communicate disease burden and provides prognostic information.^10^

### Treatment

Once the diagnosis of Marjolin's ulcer is confirmed, collaboration with a multidisciplinary oncology team will ensure that general recommendations proposed by the AJCC will be followed.^10^ Localized disease is generally managed with wide local excision with 2-4 cm negative margins.^10^,^16^, ^17^, ^18^ High-risk tumors with extensive soft tissue or bone involvement, or locoregional lymph node spread may be best managed with amputation. Distant metastases may require resection, intensity-modulated radiotherapy, or systemic chemotherapy as determined by the multidisciplinary oncology team^9^. Routinely-scheduled surveillance studies are monitored by the oncology team.

Our case report presents several unique features to the Marjolin's tumor. This mass developed as a consequence of long-standing periprosthetic joint infection in an immunosuppressed host, with a latency period of only 7 years, and an even shorter period of off-and-on wound drainage. Furthermore this malignancy developed in an intraosseous and intra-articular location, without superficial skin involvement. The case patient was fortunate, in that her locoregional lymphadenopathy was reactive in nature secondary to infection, and not consistent with metastatic spread. Ablation of the extremity was therefore potentially curative.

## Conclusion

Marjolin's Ulcers are frequently aggressive tumors complicating chronically infected wounds and tissues of the extremities. To our knowledge, the incidence of malignant transformation in chronic periprosthetic joint infection is unknown. This unusual complication was identified intraoperatively by the recognition of a tissue bed with an atypical appearance, which highlights the importance of maintaining a high index of suspicion when managing chronic musculoskeletal infection. Consistent with the adage “culture what you biopsy and biopsy what you culture,” this case confirms that it is prudent to send material for histopathologic examination when performing debridement procedures, particularly when the appearance of the wound bed or drainage have changed.

## Figures and Tables

**Figure 8:**
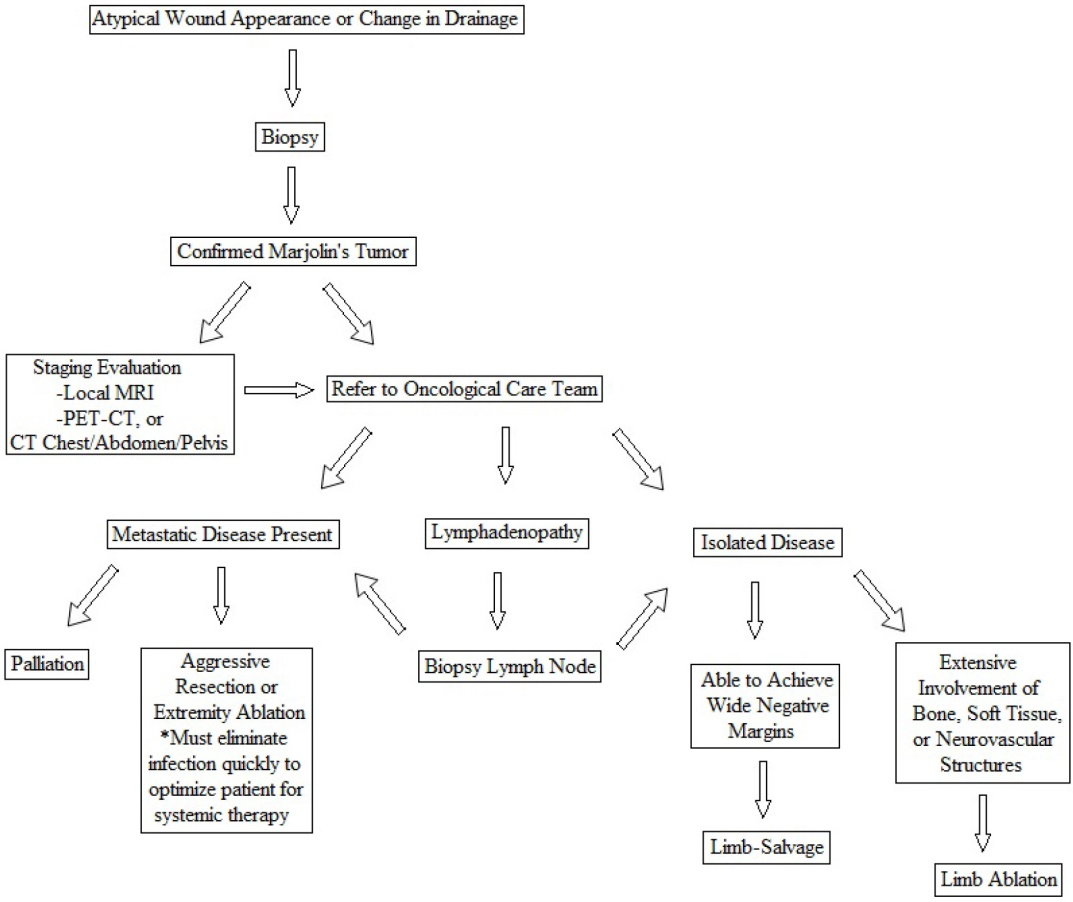
Algorithm for diagnosis and management of Marjolin tumor.

**Table 1:** 

Pathogenesis Of Marjolin's Ulcer	
Chronic irritation theory ^13^	Repeated re-epithelialization induced carcinogenesis.
Traumatic epithelial grafting theory ^15^	Skin grafts into the dermis prompt an immunological response impairing physiologic healing.
Cocarcinogen theory ^17^	Chemical toxins/physical injury stimulate latent malignant cell proliferation
Initiation and promotion theory ^15^	Two-stage process. Initiation phase where healthy cells turn into latent malignant cells. Promotion phase where malignant cells proliferate due to cocarcinogens, infection, etc.
Immunological theory ^16^	Sequestration of wound leads to avascularity and impaired immune surveillance leading to unregulated cell growth.
